# Newspapers and the Doctors

**Published:** 1893-11

**Authors:** 


THE NEWSPAPERS AND THE DOCTORS.—We hope all our readers who read our editorial on this subject in the TEXAS MEDICAL JOURNAL for July, referred to in Mr. Rider-Taylor's article under same caption, published elsewhere, will read Mr. Rider-Taylor's rejoinder, and then—read this,—the only comment we shall make. It is taken from that live and vigorous little weekly, the *Lancet-Clinic.* It will be remembered that in our last we referred to the fact that Amick's advertisements were now appearing in the press dispatches as "pure telegraphic news."

That the press should prostitute its telegraphic columns to such base uses is proot enough of the correctness of our position in the July number,—that "the press is inimical to the regular profession and favors quacks because they advertise:"

"ADVERTISING A FINE ART—THE WAY THEY DO IT.—The following is from a printed proof, pasted on a Western Union telegraph blank, and mailed to three hundred daily papers:

" 'N. Y., October 17, 1893.

" 'To the Anzeiger:

" 'Insert with striking headlines as pure telegraphic news, Wednesday sure, the following dispatch. Both items must appear under one heading. This is important. Please give these items special attention. " 'GEORGE E. GUERRIER.

" 'PHILADELPHIA, Oct, 17.—The County Medical Society's petition to the Board of Health to isolate Consumptives has increased their fears occasioned by startling head-lines in a local paper declaring the disease infectious. The State Legislature of Michigan recently endorsed this view, as did the Medical Congress in Washington, and deaths from Consumption having decreased everywhere recently. Dr. Fleck, with a few others, ascribes this to isolation. The majority of medical experts, however, credit it to the free broadcast distribution through physicians of test outfits of the Amick treatment, by which authentic cures are reported daily in the medical and secular press.' "

" 'MINNEAPOLIS, Oct. 17.—Recent local editorials condemning the medical code while commending Amick, the Cincinnati scientist, for withholding his Consumption cure formula are exciting much discussion in medical circles. The *Times* said "his discovery greatly assists the fight against this enemy of human life, and thirty or more local physicians say the medicine accomplishes more than his claim." The *Journal* said "it is one of the most valuable and wonderful discoveries ever hoped for in medical science, and the formula is not given to every Tom, Dick or Harry to monkey with, and is preserved from the tampering of fool empiricists." ' "

